# Community and School-Based Health Education for Dengue Control in Rural Cambodia: A Process Evaluation

**DOI:** 10.1371/journal.pntd.0000143

**Published:** 2007-12-05

**Authors:** Sokrin Khun, Lenore Manderson

**Affiliations:** 1 National Centre for Health Promotion, Ministry of Health, Cambodia; 2 School of Psychology, Psychiatry and Psychological Medicine, Monash University, Australia; International Technical Assistance Group, United States of America

## Abstract

Dengue fever continues to be a major public health problem in Cambodia, with significant impact on children. Health education is a major means for prevention and control of the National Dengue Control Program (NDCP), and is delivered to communities and in schools. Drawing on data collected in 2003–2004 as part of an ethnographic study conducted in eastern Cambodia, we explore the approaches used in health education and their effectiveness to control dengue. Community health education is provided through health centre outreach activities and campaigns of the NDCP, but is not systematically evaluated, is under-funded and delivered irregularly; school-based education is restricted in terms of time and lacks follow-up in terms of practical activities for prevention and control. As a result, adherence is partial. We suggest the need for sustained routine education for dengue prevention and control, and the need for approaches to ensure the translation of knowledge into practice.

## Author Summary

Health education is essential for the control of diseases such as dengue, ensuring that community members understand the mechanisms of infection and the key behaviours or activities that need to be addressed to prevent transmission, reduce severe disease and avoid fatalities. In Cambodia, health education for dengue control is provided in primary schools, at village health centres and by the National Dengue Control Program. However, these educational programs are accorded low priority, strategies and materials are not evaluated on a routine basis, messages are sometimes confusing, and health staff and teachers lack the training, time and opportunities to deliver educational messages. Recommendations to villages are not always practical or effective in preventing mosquito bites, and funds are not available to provide new educational materials. While school children and their parents therefore have some familiarity with the behaviour and habitat of the aedes mosquito and the environmental factors that contribute to dengue fever, their knowledge is uneven and knowledge is rarely translated to reduce the risk of infection. Community involvement in the prevention and control of dengue is essential, but will not be effective while health education is poorly resourced and irregular and lessons on prevention do not result in action.

## Introduction

Dengue fever (DF) and its potentially fatal forms – dengue hemorrhagic fever (DHF) and dengue shock syndrome (DSS) – are significant public health problems in tropical and sub-tropical regions worldwide. In Cambodia, dengue is particularly prevalent in children under 15 years [1]. Over the past decade, dengue has increased in prevalence. In 2007, there has been an especially deadly epidemic, with around 34542 cases of DF, DHF and DSS and 365 deaths nationwide from January to August; in the study province in eastern Cambodia, dengue was especially prevalent, with 5105 cases and 65 deaths recorded for this period [2]. The main control activity of the National Dengue Control Program (NDCP), located in the capital city, Phnom Penh, is the distribution of temephos and insecticide spraying [3]; this is undertaken primarily during the rainy season from May to October in areas which, on the basis of available morbidity statistics, are considered to be experiencing epidemic outbreaks. Temephos distribution is supported by health education, which addresses both disease prevention and early diagnosis and treatment, and is delivered within communities at health clinics, during village outreach activities and in schools. General behaviour change strategies, including in relation to larvae and vector control and early treatment seeking, and the health education materials to support these strategies, are produced by the Health Education Unit (HEU) of the NDCP, with input from various government departments.

The research literature on dengue health education indicates the effectiveness in establishing good knowledge of dengue recognition, prevention and control methods, although not necessarily in changing behaviour. A study conducted in Colima, Mexico, illustrated that continuous health education reduced the *aedes aegypti* habitats more effectively than larvicide distribution alone, or a combination of larvicide and a targeted health education campaign [4]. However, others argue that the translation of knowledge to practice varies [5]–[11]. Leontsini and colleagues [12], for instance, found that while people did translate knowledge to practice with regard to waste, the effects were limited for water storage containers. This in part related to the ineffectiveness of the methods promoted for cleaning water containers, and once this was addressed, cleaning increased [13]. Thus while preventive activities tend to be practiced most extensively by people with good knowledge of dengue [14], where educational information is insufficient to address people's understandings of disease transmission and/or the methods promoted are flawed, then people may reject specific recommendations to reduce the risk of infection [9]. Further, even with good knowledge, people may resist household or personal practices to control the vector and see such actions to be a government rather than a personal responsibility [15],[16]. This has also been reported in Myanmar, where caregivers have been reported to have good knowledge of DF transmission, but this was not translated into dengue prevention and control activities [17].

School-based education is an important compliment to community education because of the presumed transfer of knowledge and practice from classrooms to homes, because the disease predominantly affects children and because control measures such as source reduction require ongoing household activities [18]. In Puerto Rico [19] and Thailand [20],[21], primary school programs have succeeded in increasing children's knowledge of and participation in the prevention and control of DF. In Puerto Rico, school programs have resulted in good knowledge of prevention and control measures and increased participation in control activities, leading to slightly lower indices of mosquito infestation [19]. Similarly, a school-based dengue control program in Honduras increased knowledge of both the cause of dengue and the vector life cycle, leading to increased participation in controlling larval breeding sites and the consequent reduction of the number of sites [22]. However, a study among university students in the Philippines showed that the students had good knowledge of the types of mosquito larval habitats, but limited participation in source reduction activities [23].

**Figure 1:**
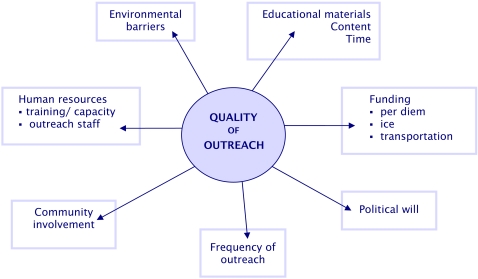
Framework for health outreach activities in Cambodia.

Health belief and behavioural change models have been used as a basis to develop educational materials to encourage community participation in the prevention and control of DF [24]. However, while the research literature describes specific interventions, little work has been undertaken on the mode of delivery or effectiveness of routine programs. Adequate funds, technical and human capacity, cooperation between health centres (HC) and village networks, and strategies for overcoming adverse physical conditions, are all necessary for effective and efficient outreach (Figure 1). Two additional factors are likely to impact on outreach activities: political commitment and the availability of educational resources. Here, we explore the input, process and effectiveness of community outreach, HC and school-based education for dengue control to address this gap. In doing so, we elucidate the challenges facing dengue prevention and control in Cambodia.

## Methods

This paper aimed to evaluate processes included in the dengue control program in Cambodia. We draw on data collected from March 2003 to February 2004 in the province of Kampong Cham (KPC), eastern Cambodia, where the NDCP was active. Dengue has a higher prevalence in this area than elsewhere in the country and is increasing: during the study period 2002–2003, there were 3713 cases of DF, DHF and DSS and 49 deaths in KPC; in the first eight months of 2007, as noted above, there were 5105 cases and 65 deaths, with around 90% of cases of disease affecting children under 15 years of age. Ethnographic research was conducted in two rural villages with the highest prevalence of the disease compared to other villages in the province. The two villages located approximately 30 km in each opposite directions of the provincial town center, around 100 km from the capital city Phnom Penh. Most villagers are poor farmers, growing rice for subsistence and sale, and supplementing this with the sale of agricultural produce and non-food goods purchased for resale from the provincial capital or Phnom Penh. Environmental conditions in the villages are conducive to dengue transmission. Coconut trees are abundant and coconut shells, plastic bags and other disposable items are indiscriminately discarded throughout villages, providing ideal conditions for the breeding of the vector, *Aedes* mosquito; water jars are rarely covered and contain larvae year round; houses are built above the ground of wood, bamboo and thatch, and are in very poor condition, allowing easy access of the anthropophilic and endophilic vector.

Ethnographic methods were employed in the study and included key informant interviews, focus group discussions, in-depth interviews and open-ended questionnaires, and observations. Four key informant interviews were conducted with village health volunteers in the research villages about villagers' awareness and perceptions about dengue and the various prevention and control dengue activities implemented in the villages. Four focus group discussions with mothers/caretakers of children infected with dengue provided a means to gain preliminary knowledge of their understanding of dengue and its prevention and control measures, and informed the range of questions explored through other methods. Twenty nine in-depth interviews were conducted women whose children had been infected in the past year or during the research period about knowledge and practices of dengue, their personal experiences of dengue infection, sources of and access to dengue health education, their participation in dengue prevention and control activities, and their views about control program activities which they may have been reluctant to discuss in front of others. These data were supplemented by questionnaires with 38 women (i.e. 19 families per village, approximately 15% of the village population) whose children had no history of dengue, on knowledge of DF and sources of DF health education. Sixteen interviews were conducted with managers and staff at HC, provincial and national levels, focusing on the planning, implementation, monitoring and evaluation of dengue health education activities. Primary school children from schools attended by children from the research villages participated in four focus group discussions and 63 open-ended exercises; again these explored knowledge and practices of dengue and its control. Each focus group discussion took around 1 hour and a half, and in-depth interviews took about an hour. Observations were conducted at the time of the interviews for evidence of practices of covering water jars, the presence of Abate bags in water jars, discarded containers, and using mosquito nets in the day time. Finally, thematic analysis was conducted of the dengue health education materials produced by the NDCP, and the curriculum materials provided to schools were analyzed for content. All qualitative data were entered into computer, codes were developed, and the data were manually analysed thematically.

Ethics approval to conduct this research was granted by the Human Research Ethics Committee of The University of Melbourne (Australia), the Ministry of Health (Cambodia), and WHO/TDR (UNICEF-UNDP -World Bank-WHO Special Programme for Research and Training in Tropical Diseases). Consent to conduct fieldwork in the two study villages was obtained from the Provincial Health Department and Municipality of KPC. Potential participants, including key informants and villagers who might participate in focus group discussions or in-depth interviews, were provided with a plain language Participation Information Sheets in Khmer, and the project was explained to them verbally. If the person agreed to participate, an appointment was made for the interview or a time set for the focus group, and at the time of data collection, consent was established again. Because the collection of signatures hold negative connotations for most people, consent was given verbally and recorded by the first author. The consent to visit the schools in the villages was obtained from the Provincial Education Department of KPC. Consent to conduct interviews, focus group discussions and observations in the individual schools were then obtained from the school principals and teachers-in-charge, who were provided with a plain language Participation Information Sheet in Khmer and to whom the project was explained verbally. Parents also provided permission for their child or children to participate, and children and teachers gave consent verbally again before commencing the interviews.

## Results/Discussion

### Health outreach activities in Cambodia

In Cambodia, no specific model of change was used to structure health outreach activities or health education materials; rather, the strategy and content were developed to include a wide range of activities, information, practices and behaviour regarded as important to control dengue and reduce mortality. Community health education is provided through radio, television, billboards, banners, flipcharts, posters and leaflets. While media exposure varies with the location of the population, approximately one third (28.2%) of rural and more than half (57.9%) of urban Cambodians possess televisions and 39% of the rural and 61.2% of the urban population have radios [25]. Specific health education materials to support individual, household and community strategies for dengue control are produced by the Health Education Unit (HEU). Dengue health education is mainly conducted nationally (through radio and television) and at local HCs, with content based on action plans developed by HC staff with the direction and advice of supervising Operational District (OD) staff [26]. Outreach activities from the HC are considered essential to improve people's access to important preventive and curative services. *The Guidelines for Outreach Services from the Health Centre* [27],[28] provides general guidance to organize outreach services and specifies implementation and monitoring activities.

**Figure 2:**
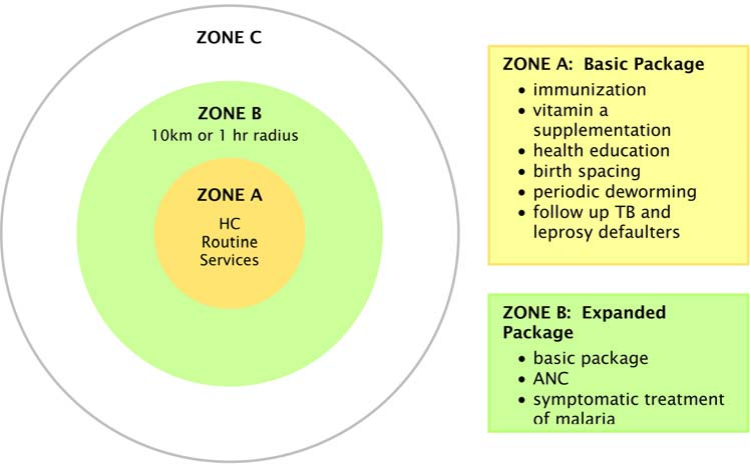
Outreach activities at health centres.

The Ministry of Health classifies HC coverage areas in terms of zones. Zone A is the village where the HC is located, Zone B comprises other villages in the HC catchment area, located less than one hour from and with easy access to the HC, and Zone C includes those villages that are located at a greater distance. Outreach varies according to zone. Both curative and preventive services are provided at the HC in Zone A. In Zone B, a basic outreach package should be provided to villages twice a month. An expanded package should be delivered to remote villages in Zone C at least six times and ideally twelve times per year. The services provided in the packages determined for the different Zones are set out in Figure 2. Both study villages in which ethnographic research was conducted were in zone B, and so subject to the first package: accordingly they should have received outreach twice a month. The outreach team, primarily midwives, should include two health workers, and activities are meant to be undertaken on a rotational basis with village health volunteers and members of village development committees. Villagers should be advised of the time and venue of the outreach before the visit.

Outreach activities are allocated the same level of financial support countrywide, with the budget integrated into the plan of action of the HC. Health staff use ministry-owned or their own motor bikes to travel to villages, and may use boats to reach flooded areas during the rainy season. The Ministry of Health finances transportation, allocates a per diem for health workers, and pays for petrol and ice for vaccine cooling, partly from the income from HC user-fees allocated for outreach to expand immunization coverage. International organizations such as UNICEF (United Nations Children's Fund), Global Alliance for Vaccines and Immunization, and UNFPA (United Nations Population Fund) also support outreach, but only for their vertical activities of interest, such as immunization coverage or family planning. No specific budget is allocated for dengue education, and so dengue outreach is included in immunization activities and covered by the immunization outreach budget.

Primarily for financial reasons, the number of outreach activities provided to villages in the study areas was much lower than that proposed by the national outreach policy. The head of one centre reported providing outreach to villages near the HC, Zone A, once a month, but villages in Zone B twice a year. No health outreach occurred in Zone C in the first seven months of 2003, because no funds were available to meet transport costs. Staff from the other HC stated that they had performed one outreach visits to each of seven villages in the first seven months of 2003. Delay in the transmission of funds to the HC was the predominant constraint. Government funds often do not reach the HC and the amount is often less than that approved. There were further delays during the 11 months of house-keeping government following the indecisive elections of July 2003, and one of the HCs received funds only for the period January to July 2003. HC health workers initially conducted outreach activities with the hope that they would be reimbursed, but over time, outreach ceased and in some cases, staff did not undertake outreach although they recorded it done.

### Content of health educational resources for the outreach

Both basic and expanded packages comprise of multiple activities, but the main activity is to provide immunization to infants and pregnant women, and the outreach is always referred to by health staff as PEV (Expanded Vaccination Program). Visual and audio health educational materials, such as posters, flyers and recordings for loud-speaker announcement, are available for immunization but also for family planning, malaria and tuberculosis. Other activities of the package are low priorities. Dengue education was conducted only when staff had time and when it was considered most salient, i.e. during the rainy season. This is despite the fact that the village environment is conducive year round to dengue transmission. For instance, coconut trees are abundant and coconut shells, plastic bags and other disposable items are discarded throughout villages, providing ideal conditions for vector breeding (*Aedes aegypti* and less commonly in this area, *Aedes albopictus*). In addition, water jars are usually uncovered and contain larvae year round, and houses are built above the ground of wood, bamboo and thatch, and are in poor condition, allowing easy access of the vector. Education related to the prevention of dengue was usually conducted verbally, without or with very limited educational material such as posters, leaflets or recorded loud speaker announcements.

Time to deliver health education, and the number of people available to receive such information, is limited. Before visiting the village, the HC is required to inform the feedback committee members in the village of the date and time of the outreach, and the venue, usually a school or temple, with committee members then informing villagers by walking house to house. Due to the lack of logistic support, the enthusiasm of feedback committee members is limited and they concentrate on their own income generating activities, limiting the extent to which villagers are informed of outreach visits. Most outreach workers from the HC travel by motorbike, carrying the immunization cool box to the village and waiting until villagers have gathered before they open the box to begin vaccinations. During this waiting period of 15 to 20 minutes, they may provide health education on topics such as family planning, HC health services and seasonal diseases including DF and diarrheal disease. When the immunization begins, one of the two health workers gives injections while the other fills in the records. After vaccination, villagers leave immediately, leaving the health workers with no further opportunity for health education.

### Access to villages

Physical access is problematic in much of Cambodia. Mountainous or remote rural areas that were former Khmer Rouge strongholds or battlefields have been underserved for years due to problems with security and landmines. Because of lack of road systems and lack of transport, many areas are isolated year round, and villages in forested areas are barely accessible. In the rainy season, the peak season for dengue transmission, flooding is common and villages become isolated. Ten of the 15 villages covered by one of the study HCs were located along branches of the Mekong River, and were flooded during the rainy season. These villages were accessible by boat, but this was time consuming and expensive: 40,000 *riels* (US$10) per trip compared with (6,000 riels or US$1.5) for travel by motor bike. Roads to other villages were very muddy, prolonging travel time and creating difficulties for HC staff.

### Technical staff competency

Health centre workers' capacity, competency, skills and experiences are crucial to the quality of preventive services in villages. Limited training was provided to them in techniques of health education, outreach strategies and dengue prevention. Staff of one HC reported receiving no training on these topics in the previous 18 months, although they had received training on immunization and family planning. Additionally, HC personnel lack the skills to identify preventive service needs or to develop and implement practical plans of action to increase coverage. Because outreach was performed on a rotational basis, so HC staff had responsibility for multiple tasks at the HC facility and for outreach and this too affected the quality of the outreach.

### Health Education Materials

#### Printed materials

**Figure 3:**
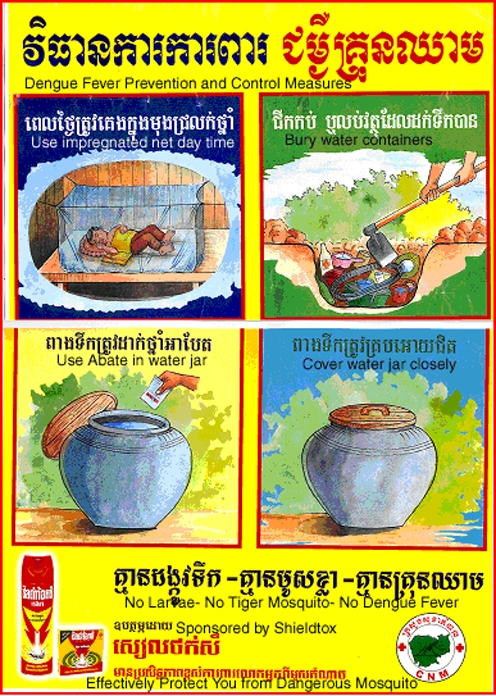
Dengue fever prevention and control measures. (Image courtesy of: National Dengue Control Program, Cambodia).

**Figure 4:**
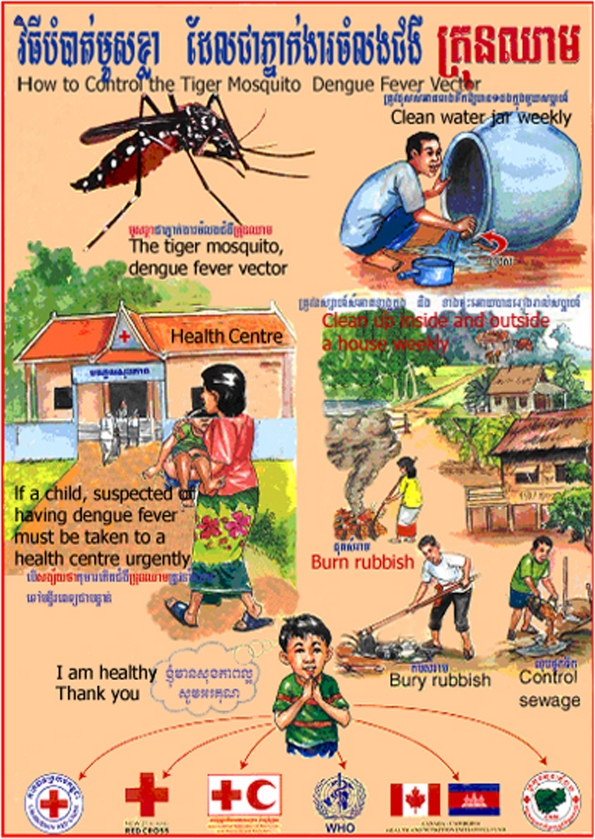
How to control the tiger mosquito dengue fever vector. (Image courtesy of: National Dengue Control Program, Cambodia).

The messages and pictures used in health education materials were developed following needs assessments conducted by HEU staff, who interviewed villagers to select appropriate messages, and modified these messages after pre-testing and post-testing with villagers, to ensure comprehensibility and clarity, before printing for posters and leaflets (see Figures 3 and 4). However, health education materials were allocated a small budget and funds for this purpose flowed irregularly, resulting in an irregular supply of materials. During the major epidemics of DF in 1998, around 10,000 posters and 30,000 leaflets were produced with the financial support of WHO; an additional 10,000 posters and 30,000 leaflets were produced with private company sponsorship in exchange for advertisements of mosquito coils and domestic insecticide spray on the materials. In 2000, materials were reprinted with International Red Cross support. Funds were allocated but not remitted from the Priority Action Program budget from 2001 to 2003, and the HEU simply distributed the materials remaining from the 2000 print run. In 2003, the HEU produced no new dengue health education materials and few dengue education activities were conducted for financial reasons.

For budgetary reasons, too, the HEU has never evaluated or tested the effectiveness of the distributed materials. As a result, the HEU still uses the messages developed in 1998, resulting in a lack of up-to-date information. The lack of evaluation has also meant that no reports were available for donors, discouraging their continued support of the program. Even with financial support, the number of printed copies was limited compared to the scope of dengue problems in the country, making it difficult for health service staff to provide dengue education within health facilities and during outreach activities. Further, during the study period printed materials were only distributed when epidemics were reported by the NDCP, and materials were not always delivered even under these circumstances; health workers reported no materials were available in 2002 despite outbreaks of infection. There was (and is) no regular distribution system to ensure that materials reach the right destination; rather, posters and leaflets may be collected from Phnom Penh when staff visited the NDCP, or distributed opportunistically when PHD/OD staff visited the HCs or when HC staff came to the OD or PHD for a meeting or seminar. Leaflets and booklets were often left undistributed in piles in the provincial health office, too, while HC had insufficient materials to distribute to people in the villages even during epidemics. Monitoring and evaluation to improve the effectiveness of the distribution of materials is not undertaken.

#### TV and Radio Spots

One to two minute spots for radio and television were produced by the HEU in 1999 and 2000 with the financial support of WHO and International Red Cross. With additional support from WHO, these spots were broadcast on three of seven television channels and four of eight radio stations country-wide. While these channels had a large coverage area, they were not the most popular; rather, they offered negotiable prices lower than those of the free market. All broadcasting was costly even on the government media network. Further, the spots were not broadcast on prime time because of the high cost of doing so. From 1999 to 2002, broadcasting was daily, but in 2003, for budgetary reasons, broadcasting lasted only for three months of the rainy seasons and was not able to produce new messages and so used spots produced in 1999 and 2000. When funds are available to the HEU and concessions are provided from media companies, then spots are broadcast routinely, but the support from media institutions, whether government-owned or privately-owned, is limited. Further, as with printed materials, for budgetary reasons, no evaluations have been conducted on people's perceptions and the effectiveness of the broadcasts.

#### The content of health education materials

Five posters, three leaflets, eight television spots and one radio spot on dengue education were collected and analyzed. Two posters were sponsored by a private company selling mosquito coils and indoor insecticide spraying, Shieldtox. The message from the company printed on the poster was “Shieldtox, the name of the coils and spray, is highly effective in preventing you from mosquitoes.” Cleaning and covering water containers were promoted on one radio spot, six television spots, four posters and all four leaflets. The same materials emphasised the importance of controlling discarded containers, included burying or burning coconut shells, cans, bottles and plastic bags, and filling sewage ponds. Temephos (Abate) was promoted in six television spots, two posters and one leaflet; insecticide spraying (other than the Shieldtox advertisement) in one television spot. Prevention from mosquito bites by using a net was highlighted in two television spots, two posters and one leaflet, and the use of mosquito coils was mentioned in one television spot. One television spot and one leaflet explained that DF could be recognized by a fever resistant to antipyretics and by other signs such as red dots on the skin and coldness at the tips of toes and fingers. The use of aspirin to reduce the fever was mentioned as inappropriate and dangerous in cases of DF in one television spot and one leaflet. Mothers were encouraged to take their sick children to a HC in three television spots, two posters and two leaflets.

#### Exposure to Health Education

Twenty four mothers of children with DF who participated in in-depth interviews were asked whether and how they heard any health education information. Women reported hearing dengue health education messages from different sources, either from the radio only, from television or from both radio and television, car (with loudspeaker). Many participants did have not a television or could not afford a battery, but they could watch television with their neighbours, only two women said they were too busy or tired to watch television after farming and were too poor to think about anything except food. One woman had heard about dengue from seeing the NDCP outreach car, mounted with a giant mosquito puppet and loudspeaker, driving through villages during the rainy season to support temephos distribution. Many women received dengue education from outreach health workers, HCs and the referral hospital in KPC, learnt about dengue from their neighbours. The majority who had received dengue education could recall at least one message, ranging from recognition of DF, the appearance of the *Aedes* mosquito (known locally, because of its markings, as tiger mosquito), biting time, breeding sites or prevention, control measures, and the importance of taking their children for attention at the HC. One mother said, “I saw it (dengue health education) on television. It told us dengue infected children and caused very high fever” (in-depth interview).

Mothers of children not infected with dengue infection were also asked whether and how they had heard dengue education messages to compare the outcomes of printed and electronic media. Most (29/38) reported hearing about dengue health education at least once during the rainy season in 2002. The majority of these women (23/29) had heard this information on radio, which they could listen to wherever they were, including when working in the fields. Many (18/29) heard messages on television, although fewer women had a television or batteries, and watching television required them to be at home. Very few women reported receiving dengue health education from health staff during outreach activities, pr when they visited the HC, the provincial referral hospital or a private health practice in the village. Only one woman heard the message from the loudspeaker, and one woman from her neighbours.

### School-Based Dengue Control Education

In 2000, the Cambodia Ministry of Education (CMoE), in cooperation with the Ministry of Health, introduced integrated dengue sessions into the biology curriculum for primary school children in grade five, together with sessions on other diseases such as malaria. Dengue was taught in a one-hour session. According to interviews with teachers, available curriculum time to cover dengue or other health topics was inadequate, and the lack of teaching materials made it difficult for them to transfer the messages from the lesson into practical actions in the villages. Teachers therefore felt that they lacked technical and logistic support, but also that they were not sufficiently trained to teach the subject. Moreover, they did have enough time to teach all the health topics in the curriculum, irrespective of their motivations, due to low government salaries: like other employees, they supplemented their salaries with subsistence production or other income generating activities.

#### Knowledge of causes and disease recognition

The students in both primary schools serving the two study villages had good knowledge of the cause of DF and vector identification. The majority (46/63) knew that DF was caused by tiger mosquito bites, most could identify the vector as having distinctive black and white markings, many students said that DF was caused by a virus, and half specified that only the female vector could transmit the virus. However, other students did not know the cause of dengue; a few believed that the disease was caused by being bitten by the *Anopheles* mosquito (“nail mosquito”) or – without linking the disease to a vector - from sleeping without a net.

Good knowledge of the signs and symptoms of DF is crucial to recognizing the disease and to seeking appropriate health care. Most students knew fever as the prominent sign of the disease. They described the fever as high – 40 to 41°C – and of one-week duration, with small red spots on the skin appearing as a sign of DF. Some children had particularly good knowledge of severe DF, which they described as including cold extremities, pale face and low blood pressure. However, a significant minority (16/93) did not know of any signs of DF.

#### Knowledge of prevention, control and care

Knowledge is crucial to identify breeding sites and participate in control activities. Most children in both schools reported that mosquito bites could be prevented by sleeping under a net, and a few mentioned the need to use nets day and night – a practice that would be impractical other than for infants. Others mentioned avoiding “playing in dark places,” such as under the house where mosquitos might be present. A few students had misconceptions of how to preventing bites, such as using a fire at night, or they could not identify any ways of avoiding being bitten; wearing long sleeved shirts was not mentioned. Both groups of students were aware of the need to control larvae, half mentioning the need for weekly cleaning of water containers, and many the need to cover containers. Students also mentioned source reduction activities, including controlling discarded water containers such as tires, cans, coconut shells, bottles and rubbish, and using Abate to control larvae. However, one in six children did not know how to control larvae. Children also emphasised the importance of promptly seeking care for a person suspected to have dengue fever, specifically that the person should be taken to a health facility for medical treatment. Again, however, one out of five children did not know where to seek care.

Focus group discussions yielded similar results. Students could describe DF transmission and reported undertaking activities, such as controlling breeding sites, to prevent disease transmission at home. However, discarded containers were abundant in house yards and schoolyards. The container and larval checklist in the school yard in one village included two water jars without covers, 15 plastic bags and 12 bottles; in the other village, there were 200 plastic bags, 15 cans and 20 coconut shells in the school yard. These containers were dry because it was dry season, although three cement baths in one school had around 200 larvae. The presence of discarded containers and other rubbish in the school yards suggested that students did not transfer prevention and control knowledge into practice, consistent with a study in Thailand where primary school children had high knowledge of DF, but only a few converted it into practice [20].

### Recommendations and concluding remarks

Health education is a critical component of community health to ensure child survival. Health education plays a major role to inform and encourage people to be responsible for and participate in preventing and controlling DF, as for other transmissible diseases. However, in resource-poor countries, the efficacy of health education is complicated by economic, political and infrastructural factors. As currently provided in Cambodia, dengue health education delivered through health outreach activities and school-based programs is insufficient, under-funded and irregular. Moreover, existing dengue health education messages, materials and strategies must be redefined to be practical, applicable and relevant.

There is little training in health education techniques for health practitioners either during their professional training or through in-service training, and therefore, they lack skills and tend to perceive that health education is not part of their work. According to interviews with mothers and as indicated through observations, health practitioners give limited dengue health education during clinic consultations or hospitalization, focusing rather on diagnosing the child presented to the clinic and on establishing methods of home treatment to reduce fever. Health education materials encourage women to use mosquito nets, coils and long-sleeve clothes, but these methods are neither practical nor effective to prevent bites for several reasons. First, *Aedes* mosquitoes bite during the day when the use of nets is impractical except for infants. Second, most women used the nets for their younger children, but DF also affects older children, adolescents and to a lesser extent adults [29]. Third, it is difficult to use the nets properly to prevent mosquitoes from entering, and most mothers placed their children on old thatched mats to sleep on floors made of bamboo sticks or wood, so mosquitoes can bite from beneath. Many women simply cannot afford nets. Similarly, mosquito coils were costly and the diffusion ineffective because houses are open, made of thatch or wood, and breezes remove it quickly. Most children play under the house and in the yard by day, too, so the coils had no effect in protecting children from bites. Finally, advocating long sleeved clothes was ineffective because children resisted being fully covered when playing. Therefore, health education messages promoting the use of nets, coils and long-sleeved clothing proved of limited value.

Most educational materials emphasized the importance of covering and cleaning water jars to prevent mosquito breeding. Yet this was often considered impracticable, as various container lids were made of wood or cement, heavy and hard to move, and others used cooking utensil lids which fitted water jars imperfectly. Wooden lids also tended to loose shape when it is exposed to the sun, leaving holes for mosquitoes to enter. Even with light lids made from metal trays or cloth, children in particular were careless about replacing them properly [30]. In Cambodia, a field trial of the long lasting deltamethrin-treated polyester netting (Permanet) proved effective to prevent not only *Aedes* adult mosquitoes from entering water jars, but would also kill newly emerging adults and kill or deter gravid females to oviposit in them [31]. However, this trial needed significant community education to ensure local involvement and the affordability of this program, and whether or not the intervention could be replicated as a standard control program is questionable. Finally, while Permanet will prevent mosquito breeding in water jars, discarded containers will remain a continued source of breeding and infection [3].

The difficulty in most health education development is the message content and mode and frequency of delivery. Messages need to be relevant to people' daily practices, and offer practical and effective activities. This is relevant to dengue prevention and control as to other diseases. As described above, between three and five messages were included in each type of educational material, including the importance of covering and cleaning water jars, removing discarded containers, using Abate, using nets and taking sick children to hospital. The number of messages resulted in recall of only selective facts and activities, suggesting the need for their reduction and prioritization. As the supply of distribution of temephos was costly, undertaken on government initiative rather than by householders, and had limited effect in the prevention and control of larvae [3], its use in particular should be excluded from the material.

Cambodia is a resource-poor country, with a critical lack of financial and technical resources to develop teaching materials and to motivate teachers. The school curriculum gives limited attention to preventive activities, such as the simple measures required to prevent and control DF. In consequence, while students had good knowledge of the vector and biology, knowledge was not linked to practice, such as by conducting routine cleaning-up activities in the school yard. *Aedes* mosquito and larvae were present in water jars and discarded containers in schools. Identifying breeding sites and larvae, and carrying out appropriate control activities, would consolidate learning and support students to undertake these activities at home too. This approach would be more effective and less costly than the didactic classroom-based one. For example, school children, with teachers and family members, might go into villages near the school to identify potential breeding sites and to clean the area of discarded containers.

Dengue is of continued importance in Cambodia and elsewhere, in rural as well as urban areas, with the risk of transmission exacerbated through population mobility and the lack of appropriate disposal of consumables and packaging. As van Damme and colleagues [32] have recently illustrated, treatment of the disease causes significant, often catastrophic, out-of-pocket household expenses. Even in wealthier countries in the region, such as Thailand [33], dengue prevention and control activities place considerable pressure on governments and householders. In Cambodia, there is high awareness among policy makers of the high costs of dengue fever on health services, governments and households [34], but at the same time, lack of financial and human resources mean that centralized control activities are unsustainable. Research conducted in northern Thailand draws attention to the complexity of links between knowledge and practice [14: 998], such that in some cases disease control practices may be greater than reported knowledge. This should not be taken as evidence of a reduced need for community education, however. Local prevention and control activities, involving community members and supported through health education within the community and in schools, offers potential to reduce the prevalence of dengue, but as we suggest in this article, this cannot occur where health education is poorly resourced and episodic, and where attention is not paid to the translation of knowledge to practice.
